# Heat Exhaustion and Heat Stroke Among U.S. Active Component Service Members, 2021–2025

**Published:** 2026-05-20

**Authors:** Alexis L. Maule, Katherine S. Kotas, Kiara D. Scatliffe-Carrion, John F. Ambrose

**Affiliations:** Disease Epidemiology Program, Defense Centers for Public Health–Aberdeen, Defense Health Agency, Aberdeen Proving Ground, MD

## Abstract

In 2025, the unadjusted incidence rates of heat stroke and heat exhaustion among U.S. active component service members were 39.4 and 170.0 cases per 100,000 person-years, respectively. The rate of heat stroke increased in 2024 and 2025, after declining for 3 years (2021-2023) at the beginning of the 5-year surveillance period. In contrast, the rate of heat exhaustion decreased in 2025, following a 4-year increase from 2021 through 2024. In 2025, male service members experienced higher rates of heat stroke, when compared to their female counterparts. Female service members and non-Hispanic Black service members experienced higher rates of heat exhaustion than their male counterparts and service members of other racial and ethnic groups, respectively. Consistent with prior annual reports, heat illness rates remained the highest among those younger than age 20 years, Marine Corps and Army members, and recruit trainees. To protect the force and increase readiness, military leaders and public health personnel can implement evidence-based prevention strategies, train service members to recognize the signs and symptoms of heat illness and take early action to counter the threat.

**What are the new findings?**

The unadjusted incidence rate of heat stroke increased 6.9% from 2024 to 2025, for the second year in a row, while the unadjusted annual incidence rate of heat exhaustion fell by 9.1% in 2025, following a 4-year increase from 2021 through 2024. Locations where entry training are conducted for new Army, Air Force, Marine Corps, and Space Force personnel accounted for 40.3% of all heat illness diagnoses over the 5-year surveillance period. Training, specifically initial training upon service entry, remains a major risk factor for heat illness occurrence among U.S. active component service members.

**What is the impact on readiness and force health protection?**

Heat exhaustion and heat stroke can both be prevented through situational awareness, application of appropriate risk management strategies, and, when necessary, effective countermeasures. Units that fail to implement heat illness mitigation measures risk impeding or interrupting training programs, leading to reduced operational tempo or critical mission failure due to lost personnel and resources.

The most serious types of heat illnesses, heat exhaustion and heat stroke, are occupational hazards associated with many of the military's training and operational environments, posing potential risks for force health protection. Heat illness refers to a group of disorders that result from a disruption of thermoregulation caused by high energy expenditure (i.e., metabolic heat production), environmental heat exposure, or a combination of both factors. ^1 - 4^

Heat illness occurs within a continuum of severity, from less severe (e.g., heat cramps, rash, edema), to heat exhaustion, followed by potentially life-threatening heat stroke. ^5^ Heat exhaustion and heat stroke are reportable medical events (RMEs) in the Military Health System (MHS) to the Disease Reporting System internet (DRSi). All heat casualties that require medical intervention or result in change of duty status must be reported by U.S. Armed Forces installation public health personnel. ^6^

During or immediately following a period of physical exertion or heat exposure, specific signs and symptoms that characterize heat illnesses allow initial recognition of their occurrence in the field, and subsequent identification or diagnosis of a heat illness that should be reported. Common signs and symptoms of heat exhaustion include weakness, muscle cramps, headache, dizziness, nausea or vomiting, tachycardia, and short-term physical collapse or debilitation. Heat exhaustion is often characterized by elevated core body temperature (greater than 100.5 °F [38 °C], but not greater than 104 °F [40 °C]) with no significant central nervous system dysfunction. If central nervous system dysfunction develops (e.g., dizziness, confusion, headache), it should be mild and rapidly resolve with rest and cooling measures, otherwise the individual may be experiencing heat stroke. ^7 - 10^

Heat stroke is a debilitating and potentially life-threatening condition most frequently characterized by evidence of severe hyperthermia (greater than or equal to 104 °F [40 °C]) and central nervous system dysfunction that can include change in mental status, delirium, stupor, loss of consciousness, or coma. ^7 - 9 , 11^ Onset of heat stroke should prompt aggressive intervention featuring rapid cooling, such as cold water immersion and iced sheets. ^12 - 14^ The literature on heat stroke management indicates consensus on prioritizing cooling over transportation for further medical attention. ^11 , 13 , 15 , 16^ Cooling is prioritized because, clinically, severity of end-organ damage and increased possibility of mortality are directly related to the degree and duration of hyperthermia. ^8 , 14 , 16^ End-organ damage due to heat stroke is most frequently observed in the liver, kidneys, cardiac and skeletal muscle. ^8 , 11 , 15 , 17^

While temperature and humidity are well recognized environmental risk factors for heat illness, there are individual, occupational, and organizational risk factors that influence heat illness occurrence. Individual risk factors include lack of acclimatization, physical fitness levels, pre-existing or recent viral illness, body composition, and personal motivation to excel. ^7 , 12^ Organizational factors include type of activity, training intensity and duration, and training schedules. ^7 , 12^ These risk factors do not work independently of each other; there is literature that suggests risk factors interact to increase risk of heat illness, making it essential that military leaders and service members recognize the full spectrum of potential factors in a training or operational environment. ^18^ For example, metabolic heat production increases during prolonged engagement in strenuous physical activity, and additional exposure to environmental heat stress elevates core and skin temperatures. ^2 , 3 , 8^

Identifying high-risk service members is critical for preventing heat illness and reducing morbidity due to heat illnesses. ^19^ Early detection reduces heat illness morbidity and severity and requires educating service members and leadership on the signs and symptoms of heat illness in addition to the incorporation of physiological monitoring, managing exceptional individuals during training (i.e., establishing minimum or maximum pacing), and removing service members from high-risk events. Heat illness mitigation strategies should be implemented for individuals as well as organizations, using a tiered risk management model. ^13^ To achieve hazard reduction, progressive training, heat acclimatization, along with ensuring proper hydration, electrolyte replacement, and nutrition before training can prepare individual service members for training and operating in high heat environments. ^3 , 20^ Risk mitigation strategies that can be instituted during training activities include adherence to work and rest guidelines, modified clothing and uniform standards, individual- or group-pacing during high-risk events (e.g., timed ruck marches), climate-adapted schedules or activities, and available cooling measures (e.g., arms immersion cooling or microclimate cooling). ^13 , 14 , 20^

Surveillance of heat illnesses is necessary to evaluate whether prevention guidelines and countermeasures are working, in addition to identifying high-risk groups and activities that may lead to heat illness. Since 2011, *MSMR* has published regular updates on the incidence of heat illness among U.S. active component service members (ACSMs). This update presents summaries of heat stroke and heat exhaustion case counts, incidence rates, and locations from 2021 through 2025.

## Methods

The surveillance population for this analysis includes all individuals who served in the active component of the Army, Navy, Marine Corps, Air Force, Space Force, or Coast Guard at any time during the surveillance period of January 1, 2021 through December 31, 2025. Space Force data are only complete for 2023 through 2025.

All data used to determine incident heat illness diagnoses were derived from 4 sources: MHS Management, Analysis and Reporting Tool (M2), Defense Medical Surveillance System (DMSS), DRSi, and Theater Medical Data Store (TMDS). Heat illness cases were identified using specific diagnostic codes from the ambulatory care encounters and hospitalizations of ACSMs in fixed military and civilian (if reimbursed through the MHS) hospitals and clinics worldwide. In addition to medical encounter data, heat illness medical event reports were identified in DRSi, including information on hospitalization status (i.e., ‘yes’ or ‘no’). If a heat illness was reported in DRSi, but not found in the medical record, the case was still counted. For example, an individual could be treated in the field by a medic for a mild or non-life-threatening heat illness without a recorded medical encounter, but the case is deemed a reportable heat exhaustion because of symptoms observed in the field.

In this update, a case of heat illness was defined as an individual with 1) a hospitalization or outpatient medical encounter record with a primary (first-listed) or secondary (second-listed) diagnosis of heat stroke (International Classification of Diseases, 9th Revision [ICD-9]: 992.0; International Classification of Diseases, 10th Revision [ICD-10]: T67.0*) or heat exhaustion (ICD-9: 992.3–992.5; ICD-10: T67.3*–T67.5*) or 2) a RME record of heat exhaustion or heat stroke. ^19^ Asterisks denote that all subsequent digits or characters noted in that diagnostic code were included in the identification of ICD-10 codes (e.g., T67.3XXA).

An individual was considered a case of heat illness only once per year. If a service member had diagnoses for both heat stroke and heat exhaustion during a given year, the more severe diagnosis (i.e., heat stroke) was selected. If a service member had inpatient and outpatient encounters for heat stroke or heat exhaustion, the inpatient encounter was prioritized over the outpatient visit, when identifying hospitalized cases. Within a calendar year, if an individual had a diagnostic code that denoted a subsequent encounter (i.e., ICD-10 seventh digit ‘D’) or an encounter for sequelae (i.e., ICD-10 seventh digit ‘S’), but had no diagnostic codes indicating an initial visit (i.e., ICD-10 seventh digit ‘A’), the case was removed to avoid over-estimating heat illness cases by including those receiving follow-up care.

For health surveillance purposes, recruit trainees were identified as ACSMs assigned to service-specific training locations and basic training periods, using an algorithm based on age, rank, and time in service. Recruit trainees were considered a separate enlisted service member category in heat illness summaries by military grade. In summaries of heat illness by location, the Defense Medical Information System Identifier (DMIS ID) was used to determine installation or geographic location of diagnosis and medical treatment.

In-theater diagnoses of heat illness were identified from medical records of deployed service members whose health care encounters were documented in TMDS. Those encounters were analyzed separately, and the same case-defining criteria and incidence rules described previously were applied.

Incidence rates (IRs) were calculated as incident cases of heat illness per 100,000 ACSM person-years (p-yrs). Percent change in IRs was calculated using unrounded rates. Because reporting heat exhaustion and heat stroke cases is required, the proportion of outpatient and inpatient cases with a report in DRSi was also calculated. ^6^

## Results

In 2025, 518 cases of heat stroke occurred throughout the MHS, resulting in an unadjusted IR of 39.4 cases per 100,000 p-yrs **(Table 1)** . Recruit trainees, Marine Corps and Army personnel, and service members younger than age 20 years experienced the highest subgroup-specific IRs of heat stroke, as well as those in combat-specific occupations. Service members of different races and ethnicities had similar rates of heat stroke. The rate of heat stroke was 76.2% higher among men (42.8 cases per 100,000 p-yrs) compared to women (24.3 cases per 100,000 p-yrs). Recruit trainees experienced rates of heat stroke 3.5 and 3.1 times higher than other enlisted service members and officers, respectively.

**TABLE 1.** Incident Cases ^a^ and Incidence Rates ^b^ of Heat Illness, Active Component, U.S. Armed Forces, 2025

	Heat Stroke		Heat Exhaustion		Total Heat Illness Diagnoses	
	No.	Rate ^b^	No.	Rate ^b^	No.	Rate ^b^
Total	518	39.4	2,233	170.0	2,751	209.4
Sex						
Male	460	42.8	1,776	165.2	2,236	208.1
Female	58	24.3	457	191.3	515	215.6
Age group, *y*						
<20	77	86.3	471	527.8	548	614.0
20–24	239	61.1	966	247.0	1,205	308.1
25–29	121	39.0	443	142.9	564	181.9
30–34	55	25.5	192	89.1	247	114.6
35–39	15	8.9	112	66.2	127	75.1
40+	11	7.9	49	35.3	60	43.3
Race and ethnicity						
White, non-Hispanic	265	40.4	1,031	157.2	1,296	197.6
Black, non-Hispanic	88	40.0	441	200.2	529	240.2
Hispanic	105	38.2	474	172.6	579	210.9
Other, unknown ^c^	60	36.8	287	176.1	347	213.0
Branch of service						
Army	307	68.8	1,223	274.0	1,530	342.7
Navy	32	9.6	240	72.3	272	81.9
Air Force	19	6.1	253	80.6	272	86.6
Marine Corps	158	92.9	483	284.0	641	376.9
Space Force	1	10.1	5	50.7	6	60.8
Coast Guard	1	2.4	29	70.7	30	73.1
Military status						
Recruit trainee	36	127.1	414	1,462.2	450	1,589.3
Enlisted	383	36.8	1,622	155.7	2,005	192.5
Officer	99	40.6	197	80.8	296	121.4
Military occupation						
Combat-specific ^d^	185	115.8	515	322.4	700	438.2
Motor transport	17	39.3	63	145.5	80	184.8
Pilot, air crew	3	6.9	9	20.7	12	27.5
Repair, engineering	28	7.9	115	32.5	143	40.4
Communications, intelligence	17	6.3	38	14.1	55	20.5
Health care	25	23.9	85	81.4	110	105.3
Other, unknown	243	71.5	1,408	414.1	1,651	485.5

Abbreviations: No., number; y, years.

a One case per person per calendar year.

b Rate per 100,000 person-years.

c Includes those of American Indian/Alaskan Native, Asian, Native Hawaiian/Pacific Islander, and unknown race or ethnicity.

d Includes infantry, artillery, combat engineering, armor.

**FIGURE 1.**
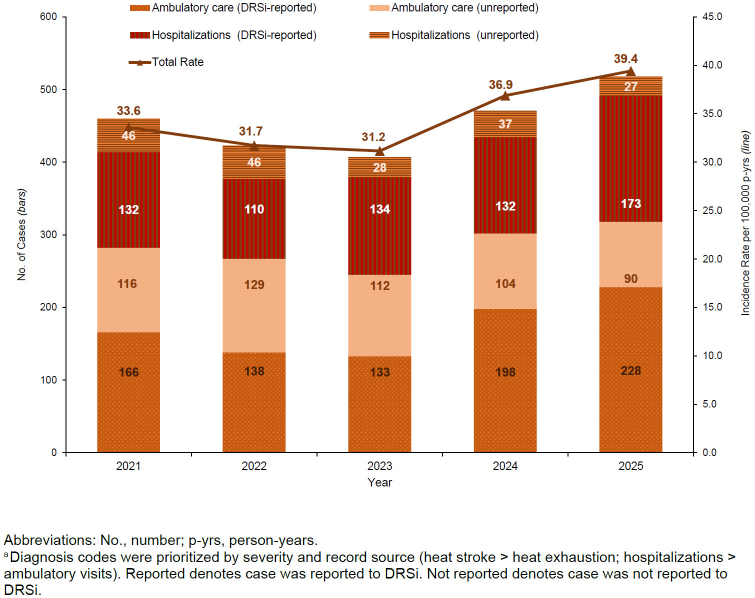
Incident Cases ^a^ and Incidence Rate of Heat Stroke, by Encounter Type and Year of Diagnosis, Active Component, U.S. Armed Forces, 2021–2025

In 2025, the unadjusted annual incidence of heat stroke increased 6.9% compared to the IR in 2024 **(Figure 1)** . In 2025 IRs of heat stroke increased among service members in the Army (21.1%) and Marine Corps (6.3%) but decreased among service members in the Air Force (-24.5%) and Navy (-51.0%) **(Table 2)** . The proportion of hospitalized heat stroke cases increased slightly, to 38.6% in 2025, from 35.9% in 2024 **(Figure 1)** . Of all inpatient heat stroke cases from 2021 through 2025, 78.7% had a medical event report in DRSi, compared to 60.5% of outpatient heat stroke cases.

**TABLE 2.** Annual Incident Cases ^a^ and Incidence Rates ^b^ of Heat Illness, by Service, Active Component, U.S. Armed Forces, 2021–2025

	Army		Navy		Air Force		Marine Corps		Space Force		Coast Guard	
	No.	Rate ^b^	No.	Rate ^b^	No.	Rate ^b^	No.	Rate ^b^	No.	Rate ^b^	No.	Rate ^b^
Heat exhaustion												
2021	1,032	215.6	147	43.0	184	53.8	494	275.4	—	—	5	12.4
2022	1,033	224.3	191	56.5	237	73.8	554	318.5	—	—	19	47.9
2023	1,179	263.1	205	62.7	363	111.0	491	290.7	7	81.9	6	15.5
2024	1,211	278.4	203	63.2	298	92.8	655	397.5	4	43.4	17	43.2
2025	1,223	274.0	240	72.3	253	76.2	483	284.0	5	50.7	29	70.7
Heat stroke												
2021	298	62.3	25	7.3	27	7.9	110	61.3	—	—	0	0.0
2022	225	48.8	31	9.2	32	10.0	134	77.0	—	—	1	2.5
2023	234	52.2	36	11.0	20	6.1	114	67.5	0	0.0	3	7.7
2024	247	56.8	41	12.8	38	11.8	144	87.4	0	0.0	1	2.5
2025	307	68.8	32	9.6	19	5.7	158	92.9	1	10.1	1	2.4
Total heat illness diagnoses												
2021	1,330	277.9	172	50.3	211	61.7	604	336.7	—	—	5	12.4
2022	1,258	273.1	222	65.7	269	83.7	688	395.6	—	—	20	50.4
2023	1,413	315.3	241	73.7	383	117.1	605	358.2	7	81.9	9	23.2
2024	1,458	335.2	244	76.0	336	104.6	799	484.9	4	43.4	18	45.8
2025	1,530	342.7	272	81.9	272	81.9	641	376.9	6	60.8	30	73.1

Abbreviations: No., number; y, years.

a One case per person per calendar year.

b Rate per 100,000 person-years.

The 2,233 cases of heat exhaustion in 2025 correspond to an unadjusted IR of 170.0 cases per 100,000 p-yrs **(Table 1)** . As with heat stroke, rates of heat exhaustion remained highest for service members younger than age 20 years, Marine Corps and Army personnel, and recruit trainees. Unlike heat stroke, however, the rate of heat exhaustion was higher among women (15.8% higher compared to men) and non-Hispanic Black service members (27.4% higher compared to non-Hispanic White service members). Recruit trainees experienced rates of heat exhaustion 9.4 and 18.1 times higher than other enlisted service members and officers, respectively.

**FIGURE 2.**
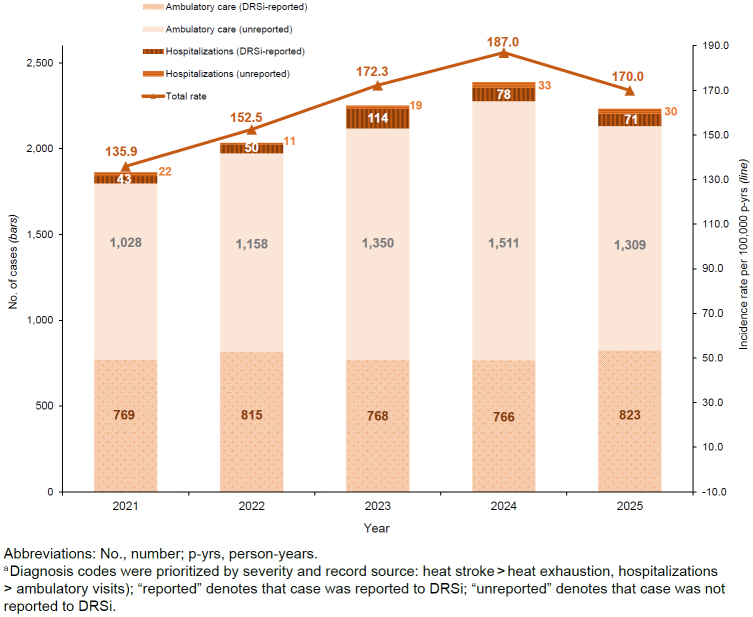
Incident Cases ^a^ and Incidence Rates of Heat Exhaustion, by Encounter Type and Year of Diagnosis, Active Component, U.S. Armed Forces, 2021–2025

After increasing in the previous 4 years, in 2025 the unadjusted annual incidence of heat exhaustion decreased 9.1% compared to 2024. Service-specific rates of heat exhaustion decreased in 2025 among Marine Corps (-28.6%), Air Force (-16.7%), and Army personnel (-1.6%) compared to the rates observed in 2024 **(Table 2)** . The proportion of hospitalized heat exhaustion cases in the U.S. Armed Forces remained small (4.5%) **(Figure 2)** . Three-quarters (75.6%) of inpatient heat exhaustion cases had reports in DRSi from 2021 to 2025, while only 38.2% of outpatient heat exhaustion cases had a medical event report.

### Heat illnesses by location

During the 5-year surveillance period, at more than 300 military installations and geographic areas worldwide, a total of 13,047 heat illness cases were diagnosed among ACSMs **(Table 3)** . Only 7.5% of those heat illness cases occurred outside the U.S., including 409 in Okinawa, Japan. From 2021 to 2025, 21 locations reported at least 100 cases of heat illness, and those 21 locations accounted for over three-quarters (75.4%) of all ACSM cases. The 4 Army installations (Fort Benning, GA; Fort Jackson, SC; Fort Leonard Wood, MO; Fort Sill, OK), 2 Marine Corps bases (Marine Corps Recruit Depot [MCRD] Parris Island/Beaufort, SC and MCRD San Diego/NB San Diego, CA) and 1 Joint Base (JB San Antonio, TX) where initial entry training occurs accounted for 40.3% of the heat illnesses during the surveillance period. Of the 21 locations with at least 100 cases of heat illness, 14 are in the southern U.S.

**TABLE 3.** Heat Injury Events ^a^ by Location of Diagnosis or Report (with at least 100 cases during period of surveillance), Active Component, U.S. Armed Forces, 2021–2025

Location of Diagnosis	No.	% Total
Fort Benning, GA	1,737	13.3
Fort Bragg, NC	942	7.2
MCB Camp Lejeune/Cherry Point, NC	829	6.4
JB San Antonio, TX	751	5.8
MCRD Parris Island/Beaufort, SC	679	5.2
Fort Campbell, KY	640	4.9
Fort Polk, LA	533	4.1
MCRD San Diego/NB San Diego	485	3.7
Fort Hood, TX	452	3.5
Okinawa, Japan	409	3.1
Fort Jackson, SC	328	2.5
MCB Quantico, VA	316	2.4
MCB Camp Pendleton, CA	313	2.4
Fort Sill, OK	290	2.2
Fort Stewart, GA	180	1.4
Fort Shafter, HI	177	1.4
Twentynine Palms, CA	177	1.4
Fort Irwin, CA	175	1.3
Fort Leonard Wood, MO	162	1.2
NAS Pensacola, FL	137	1.1
Fort Bliss, TX	127	1.0
Outside the U.S. ^b^	573	4.4
All other locations	2,635	20.2
Total	13,047	100

Abbreviations: No., number; MCB, Marine Corps Base; MCRD, Marine Corps Recruit Depot; NAS, Naval Air Station; NB, Naval Base; JB, Joint Base.

a One heat illness per person per year.

b Excluding Okinawa, Japan.

Note: Initial entry recruit training locations include Fort Jackson, Fort Leonard Wood, Fort Benning, Fort Sill, MCRD Parris Island/Beaufort, MCRD San Diego/NB San Diego, and JB San Antonio. Fort Polk is the Joint Readiness Training Center (JRTC) and Fort Irwin is the National Training Center (NTC).

### In-theater diagnosis of heat illness

**FIGURE 3.**
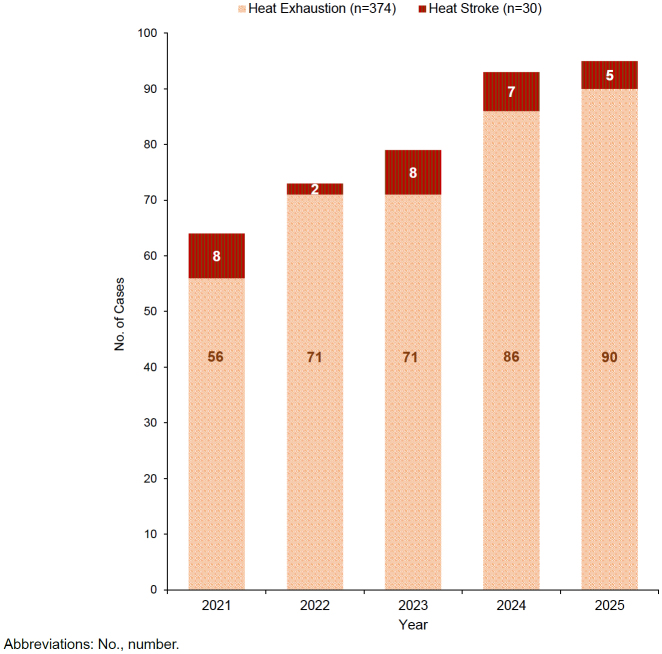
Incident Cases of In-Theater Heat Illnesses, Active Component, U.S. Armed Forces, 2021–2025

During the 5-year surveillance period, 404 cases of heat illness occurred in-the-ater, with the highest number reported in 2025 **(Figure 3)** . Heat stroke cases accounted for 7.4% (n=30) of those 404 cases of heat illness. Cases of heat illness occurred most frequently among deployed ACSMs who were male (n=295, 73.0%), ages 20-24 years (n=194, 48.0%), and in the Navy (n=230, 56.9%) (data not shown).

## Discussion

During the 5-year surveillance period, the rate of total heat illness diagnoses increased annually from 2021 through 2024 and then decreased by 6.4% in 2025. The 2025 decrease was driven by a 9.1% decrease in the rate of heat exhaustion among ACSMs in 2025 compared to 2024. The decreased IR of heat exhaustion was most prominent among Marine Corps and Air Force ACSMs. The rate of heat stroke increased by 6.9% during the same period, however, with rising IRs among Army and Marine Corps personnel.

To support the surveillance of heat illnesses among the U.S. Armed Forces, reporting heat exhaustion and heat stroke cases to DRSi is required. ^6^ As in previous reports, the proportion of heat exhaustion cases reported in 2025 to DRSi was sub-stantially lower than the proportion of heat stroke cases reported (40.0% versus 77.4%, respectively). The proportion of heat stroke cases being reported to DRSi continues to improve, however, with the highest frequency of cases reported in 2025. The *Armed Forces Reportable Medical Events Guidelines and Case Definitions* provides military Preventive Medicine and Public Health departments with the criteria for reporting these cases to DRSi. ^6^

There are limitations to this update that should be considered when interpreting its findings. Although heat illnesses were summarized by the location of diagnosis or report, medical care may not occur at the same location (i.e., installation or base) as the heat illness event, particularly if the case required a level of care not available locally. To account for locations with medical care redundancy, some installations were combined (e.g., MCB Camp Lejeune / Cherry Point, NC in **Table 3** ); this merging of locations was most prevalent with Marine Corps and Navy locations. Further, the method used to identify recruit trainees likely resulted in some misclassification of recruit training status. The algorithm did not account for the additional training time in the Army's One Station Unit Training beyond the traditional basic combat training period and does not account for service members who are recycled through training, likely leading to an under-estimation of the heat illnesses among recruit trainees. Finally, there was likely incomplete capture of heat illnesses treated in the field during training and deployments, rather than at a fixed military hospital or clinic; this may be particularly true for heat exhaustion cases when symptoms rapidly resolve after a period of rest.

Heat illness surveillance helps military public health and leadership understand the impact these conditions have on service member health, training, and force readiness. To mitigate the personal and organizational impacts of heat illness, leaders, training cadres, and supporting medical and safety personnel must inform both their subordinate and supported service members of heat illness risks, preventive measures, early signs and symptoms of illness, and appropriate interventions. To preserve readiness and protect military personnel, Department of War standards, policies, or guidelines should support heat illness surveillance coupled with evidence-based prevention, mitigation, and management practices.
